# Beyond the Transformer: A Novel Polynomial Inherent Attention (PIA) Model and Its Great Impact on Neural Machine Translation

**DOI:** 10.1155/2022/1912750

**Published:** 2022-09-21

**Authors:** Mohammed ELAffendi, Khawlah Alrajhi

**Affiliations:** Department of Computer Science, Prince Sultan University, Riyadh, Saudi Arabia

## Abstract

This paper describes a novel polynomial inherent attention (PIA) model that outperforms all state-of-the-art transformer models on neural machine translation (NMT) by a wide margin. PIA is based on the simple idea that natural language sentences can be transformed into a special type of binary attention context vectors that accurately capture the semantic context and the relative dependencies between words in a sentence. The transformation is performed using a simple power-of-two polynomial transformation that maintains strict consistent positioning of words in the resulting vectors. It is shown how this transformation reduces the neural machine translation process to a simple neural polynomial regression model that provides excellent solutions to the alignment and positioning problems haunting transformer models. The test BELU scores obtained on the WMT-2014 data set are 75.07 BELU for the EN-FR data set and 66.35 BELU for the EN-DE data set—well above accuracies achieved by state-of-the-art transformer models for the same data sets. The improvements are, respectively, 65.7% and 87.42%.

## 1. Introduction

Transformers are currently state-of-the-art models in neural machine translation [1, 2]. Since their introduction in 2017 [3], transformers have consistently produced state-of-the-art results in many areas of NLP and NMT [4–14]. However, based on recent results, it appears that transformers are reaching their limits. Despite the efforts made to improve performance and boost accuracy, the top accuracy remained around 46.4 BELU [15], and only in exceptional cases exceeded this level [16–37].

### 1.1. The Problem

As reported by recent works, some improvements are needed in the following areas:

- Transformers lack the inherent positioning naturally maintained by sequence-to-sequence models [27]
- Training is not a trivial task and may lead to destabilization of the model and taking it off track [18]
- There are problems with capturing the localness [28]
- There is a lack of scalability at the document level [24]
- The number of parameters is large, and training may take many hours or sometimes days [26]
- Accuracies are a function of model depth and size [26]

Faced with these problems and the apparent stagnation in ideas, we decided to stand back, take a deep look, and think out of the box. Thus, “thinking out of the box” is what we did. This paper shows how switching to the number theory domain provides a panorama of possibilities that are not available in the pure text domain. The starting point is the simple idea that the spatial (nontemporal) semantic dependencies between words in any NLP sentence can be accurately captured by a simple power-of-two binary polynomial transformation (PBPT). It is shown how PBPT is used to transform sentences into attention context vectors, and how the resulting vectors can be used in a neural polynomial regression [38] encoder-decoder model to generate translations.

The accuracy and robustness of the PIA-based encoder-decoder model have been assessed using the WMT-2014 benchmark data set. Experimentation revealed that the test accuracies obtained are between 73 and 75.07 BELU for the EN-FR set and around 66.35% BELU for the EN-DE set, well above the the state of the art accuracies: 46.4 BELU for EN-ER [26], 35.14 BELU for EN-DE [23]. The improvements are 65.7% and 87.42%, respectively, for the two data sets. The training time to achieve these accuracies is considerably lower than that of transformer models.

In the sections below, it is shown how this transformation reduced the neural machine translation process to a simple neural polynomial regression process using a novel polynomial attention scheme.

## 2. Background: NLP Neural Attention Mechanisms and the Transformer

**Figure 1:**
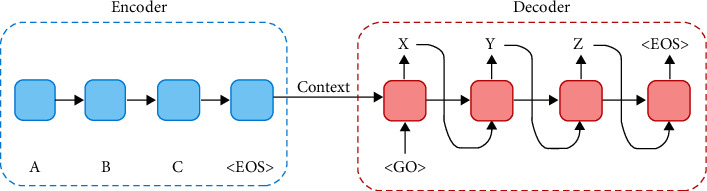
Sequence-to-sequence encoder-decoder NMT model.

Neural machine translation (NMT) is relatively a young field that emerged in 2013 as a strong alternative to statistical machine translation [39, 40]. Work in the field has been triggered by two articles: Kalchbrenner and Blunsom [39] and Stskever et al. [40]. The second article (Stskever et al. [40]) explicitly introduced the first generation NMT sequence-to-sequence encoder-decoder deep learning architecture, shown in Figure 1.

As clear from Figure 1, the encoder applies a sequential step-by-step process to encode the source sentence and incrementally computes a fixed-size context vector *C*_*t*_ that captures the temporal dependencies between sentence components. Note that *C*_*t*_, in this case, is computed using a sequential cumulative process within LSTM cells.

The context vector is then passed to the decoder where it is combined with the target sentence representation to generate the required translations word by word. The process is called “teacher forcing.”

**Figure 2:**
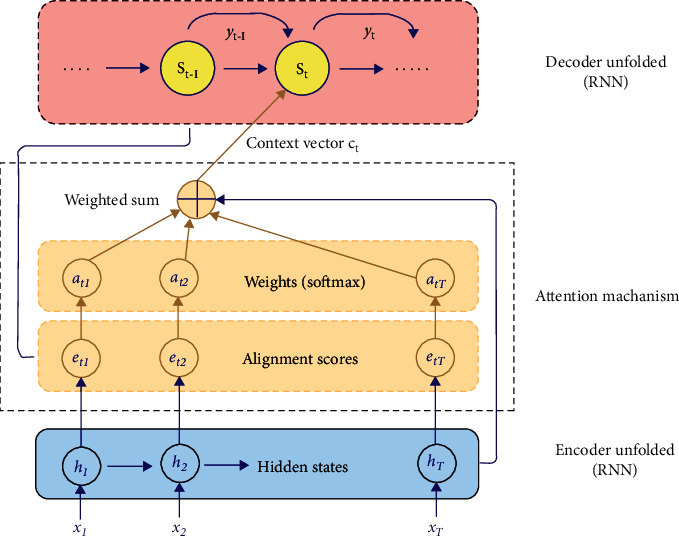
Traditional neural attention computed directly based on the hidden states.

Shortly after introducing the encoder-decoder model, it has been realized that the accuracy of the encoder context vectors fades out as a function of the sequence length, a problem that negatively impacts the translation process. This led to the emergence of the first version of NLP attention (Bahdanau et al. [41]), where a different type of context vector is computed directly using the hidden states of encoder cells (see Figure 2).

As apparent from the diagram, the traditional attention context vector is computed as a weighted sum of the hidden states *h*_i_.

This version of attention is called the “Bahdanau attention” [41] and is an example of a family of attention schemes that emerged during the same period, collectively referred to as “traditional attention.” Traditional attention led to observable improvements in accuracy, but research efforts continued to improve the accuracy and performance of NMT models.

### 2.1. The Transformer

The next important big step in the evolution of NMT attention models is the transformer, introduced by a group of Google researchers in a famous “Attention Is All you Need” paper [29]. The inventors of the transformer realized that sequential deep learning models are more of a burden rather than a booster in computing attention. The sequential step-by-step computation of the context vector is negatively impacting the degree to which the resulting vectors capture the sequential dependencies between words. The context representation fades out in accuracy as the sequence grows in length. Accordingly, the transformer designers opted for using feedforward nonsequential deep learning models and added a positioning layer to impose some artificial sequencing that emulates sequential models. Instead of applying traditional cross-layer attention schemes, the transformer applies a “self-attention” model, where attention is computed over the components of the same layer [42]. Self-attention captures the inner semantic dependencies between words in a sentence and is more appropriate in the case of feedforward neural models. As expected, these changes led to higher accuracies and better performance.

**Figure 3:**
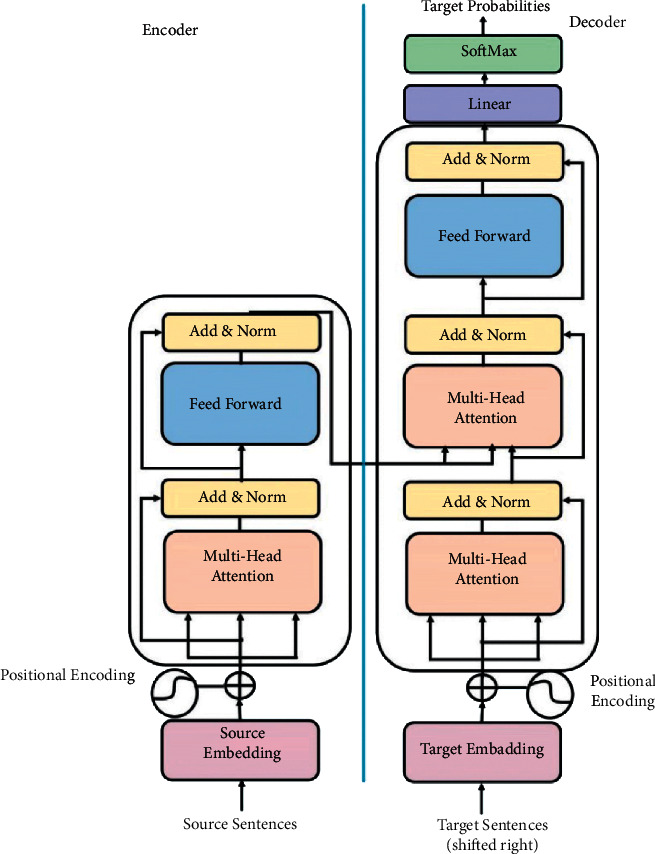
The transformer encoder-decoder model.

As shown in Figure 3, the transformer still applies an encoder-decoder architecture with teacher forcing. Both the encoder and decoder are feedforward models. The training process may be described as follows

#### 2.1.1. The Encoder

1. The encoder receives the raw representations of the source sentences in the first layer (sentence by sentence).
2. In layer 2, the encoder computes the embeddings for input sentences.
3. In layer 3, positional encoding is applied to superimpose artificial sequencing on the embedding vectors of the sentences (to emulate sequential models).
4. Multihead attention is computed in layer 4 (based on the self-attention model).
5. The normalized self-attention vectors are then passed to the feedforward network.
6. The outcome of the feedforward network is passed as input to the multihead attention layer in the neighboring decoder, where it is combined with the output of the decoder masked multihead attention layer to compute a new multihead attention layer. This is how teacher forcing is used to generate translations (see Figure 3).
7. The resulting decoder multihead attention context vector is then passed to a feedforward layer that generates the translations word-by-word.

#### 2.1.2. The Decoder

A similar process is used to handle the input target vectors on the decoder side. The operations applied include embedding and positioning, followed by masked multihead attention.

#### 2.1.3. Comments

Note that in the transformer model:

1. Both the encoder and decoder are based on nonsequential multilayer feedforward neural networks. In real applications, stacks of more than one encoder/decoder are used in the same model (commonly about six).
2. The encoder self-attention vector (multihead attention) is computed at the fourth level after the embedding and positional encoding of the source sentence.
3. The encoder attention (context) vector is then passed through a feedforward neural network to the decoder multihead attention layer to trigger the emulated teacher forcing process.
4. The translations are produced by the subsequent decoder feedforward model using a SoftMax activation function.

## 3. A Novel Polynomial Inherent Attention (PIA) Model and Its Implications for NLP

As hinted in the introduction, current NLP attention mechanisms are reaching their limits, and some new input is needed in this area. In this section, we describe in some detail a novel number-theoretic attention model based on a simple power-of-two binary polynomial transformation (PBPT). It is shown how PBPT can be used to transform any natural language sentence into a binary power-of-two big binary integer.

Since PBPT inherently preserves the positions of words in the resulting binary representation of the sentence, this representation may be viewed as an attention context vector and a unique fingerprint that captures the spatial semantic dependencies between words in the sentence.

In Section 3.1, the PBPT is described in detail showing how PIA attention vectors are generated using some illustrations.  Section 3.2 discusses the interesting properties of PIA context vectors.  Section 3.3 describes the general architecture of the PIA attention model.  Section 3.4 describes the simple power-of-two binary transformation algorithm.

### 3.1. The PIA Attention Mechanism: A Power-of-Two Binary Polynomial Transformation (PBPT)

The PIA attention mechanism is based on the simple idea that any natural language sentence can be transformed into a big integer in a power-of-two number system using a simple polynomial transformation. By “power-of-two” here we mean that the radix *x* of the target number system is a power-of-two expressed as *x* = 2^*n*^ for some integer *n*. For example, the hexadecimal system is a power-of-two number system in which the radix *x* is 2^4^. The resulting big integer is then converted to a binary vector that encodes the coefficients of the transformation polynomial.

For character-based languages, the transformation can be achieved using a two-level process:.

#### 3.1.1. Level 1: Word-Level Transformation

Transform each word *w* in the sentence into a power-of-two integer using the simple power-of-two polynomial:

(1) $$\begin{array}{c} \text{wint}=c_{0}+c_{1}.*y+c_{2}*y^{2}+c_{3}*y^{3}+\ldots +c_{m}*y^{m},\\ \text{wbin}=\text{Bin}\left(\text{wint}\right),\\ \text{where Bin}\left(\right)\text{ here referes to a binary transformation method,} \end{array}$$

where *y* is a power-of-two radix of the number system (expressed as *y* = 2^*n*^) and *c*_1_, *c*_2_,…*c*_*n*_ are integer indexes for the constituent characters based on their order in the unicode table.

Note that the actual value of the radix depends on the size of the character set. For most character-based languages, the extended character set is above 45. The closest power-of-two radix, in this case, is 2^6^, which accommodates up to 64 characters, including punctuation marks.

#### 3.1.2. Level 2: Sentence Transformation

Transform each sentence into a power-of-two integer using the simple polynomial representation below:

(2) $$\begin{array}{c} {\text{sint}=\text{wint}}_{\text{0}}+{\text{wint}}_{1}*x+{\text{wint}}_{2}*x^{2}+{\text{wint}}_{3}*x^{3}+\ldots {\text{wint}}_{m}*x^{m}\\ \text{sbin}=\text{Bin}\left(\text{sint}\right)\\ \text{pia}-\text{context}=\text{array}\left(\text{sbin}\right), \end{array}$$

where *x* is the power-of-two radix expressed as *x* = 2^*n*^, for some integer *n*; the *wint*_*i*_ variables are the power-of-two integer values computed for each *w*_*i*_ using the polynomial transformation (1). Note that the actual value of the radix in this case depends on the maximum number of characters in a word and the number of binary digits required to represent a character in the number system. Assuming that the maximum number of characters in a word is 10 and each character requires six bits, then the total number of bits in a word is 60. Hence, the radix for the sentence level number system is 2^60^.

Note that the binary vector *sbin* is the required PIA attention context vector used in later sections to generate translations using polynomial regression encoder-decoder models.

### 3.2. The Interesting Properties of the PIA Attention Vectors

The interesting properties of PIA context vectors can be summarized as follows:

1. The PIA attention vector (pia-context) is in essence a positional list of all coefficients of the transformation polynomial.
2. Since PBPT preserves the exact positions of words in a sentence, pia-context may be viewed as a spatial semantic dependency vector for words in the sentence, where the first word is the most significant digit, and the last word is the least significant digit: (see Table 1).
3. Here *wint*_*i*_ is the *i*^th^ coefficient of the polynomial that corresponds to the *i*^th^ word of the sentence and the *i*^th^ digit of pia-context.
4. The spatial (nontemporal) parallel semantic dependency property of PBPT is an alternative to the sequential semantic dependency model used in traditional attention language models.
5. From a semantic point of view, the pia-context is a unique fingerprint for the sentence. It captures the semantics of sentences and words. Accordingly, pia-context is also an embedding vector that captures the semantics of words and sentences.
6. While traditional embedding vectors are empirically computed during training using a large corpus [42], pia-context vectors are initially computed off-model before training but can evolve into other values during training without disturbing the positioning of words in the sentence.
7. It is clear that the PIA attention layer covers the functionalities of the first four layers of the transformer model (Figure 3), including embedding, positioning, and multihead attention layers.

The following simple example provides an illustration of how pia-context vectors are computed for the simple sentence (table 2 below):

Computer science department:

  Step 1: transform each word in the sentence into the power-of-two binary form:
   Computer (see Table 2)
   Science (see Table 3)
   Department (see Table 4)
  It is interesting to see how PBPT preserves the positions of characters in the binary representation of words
  Step 2:
  The binary representation of the words is then combined using a shift-left process to form the sentence binary representation (see Table 5)

### 3.3. The PIA Attention Model Architecture: “PIA Is Literally All You Need”

In the context of NLP and NMT, attention is defined as a neural mechanism used to compute context vectors that capture the semantic dependencies between words in a sentence as accurately as possible. This definition says nothing about the contents of the context vector, the type of dependency, or the way the contents are computed.

In the case of the transformer and traditional attention, the focus is on temporal sequential dependencies, and the context vectors are computed empirically during training. In such cases, the contents of the context vector are scores or weights that help the decoder generate the appropriate word translations by focusing on the relevant regions in the input vectors.

In traditional and transformer models, encoder context vectors are used to support the decoder in generating the final translations. The support model is called “teacher forcing,” where context vector scores are combined with the decoder input target words to produce the desired results.

**Figure 4:**
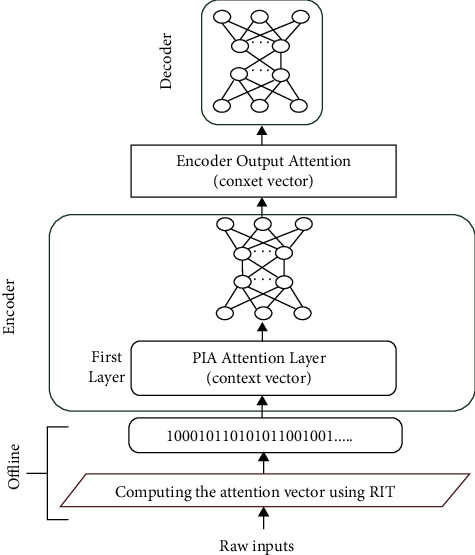
The PIA model architecture.

In the case of PIA, the following important features differentiate PIA form transformer and traditional attention models:

1. In PIA the focus is on spatial (nontemporal) semantic dependencies between words rather than temporal sequential dependencies
2. The spatial dependencies are strictly captured using power-of-two number system rules
3. This applies to all representation levels of the sentence, from the binary digit to the full word
4. PIA may be thought of as a spatial power-of-two version of the transformer self-attention approach
5. PIA context vectors are computed off-model and completely replace raw inputs and embeddings
6. As shown in Figure 4, the first layer in the PIA encoder is actually a PIA attention vector (context vector) that has been computed off-model
7. It can also be seen that the encoder first attention layer in the PIA is equivalent to the first four layers of the transformer: input layer, embedding layer, positional encoding layer, and multihead attention layer
8. Clearly, the PIA model is inherently integrated into the training neural model and is completely overlapped with it

Based on these points, the structure of the PIA model is shown in Figure 4.

### 3.4. Computing the PIA Attention Vectors

PIA attention vectors are computed using the reverse integer transformation algorithm (RIT) [43, 44]. The main RIT algorithm is described below.

#### 3.4.1. Computing the Power-of-Two Binary Representation of Words

**Figure 5:**
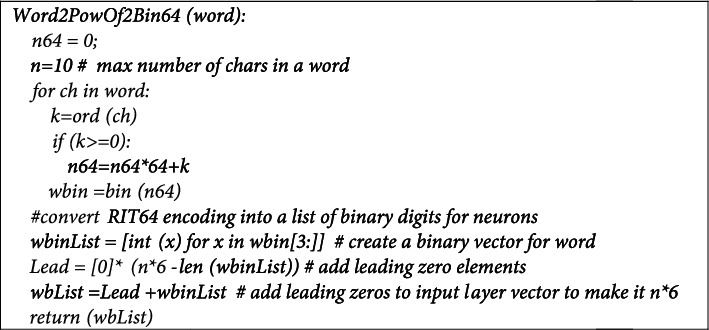
Algorithm to compute a power-of-two representation of a word.

The algorithm shown in Figure 5 below is used to convert words into power-of-two binary form. The algorithm assumes that the radix is 2^6^, which implies that each character will be represented by exactly six bits, and the alphabet to represent the words can include a maximum of 64 different characters, including the basic characters in the language.

Note that the above method assumes that the maximum number of characters in a word is 10. Since each character is represented by 6 digits, the size of a word binary representation is 60 bits. The max 10-char limit assumption is based on experimentation with different options.

The power-of-two binary representation of sentences is computed using a simple iterative shift-left process where the binary representation of each word in the sentence is appended to the right side of the sentence (a shift-left implementation of the algorithm in Figure 5). We start with an empty list and build the power-of-two sentence binary representation as follows:

BintSent = 
For the word in a sentence
    wbin = Word2PowOf2Bin64(word)
    BinSent.shifleft(60, wbin)

**Figure 6:**
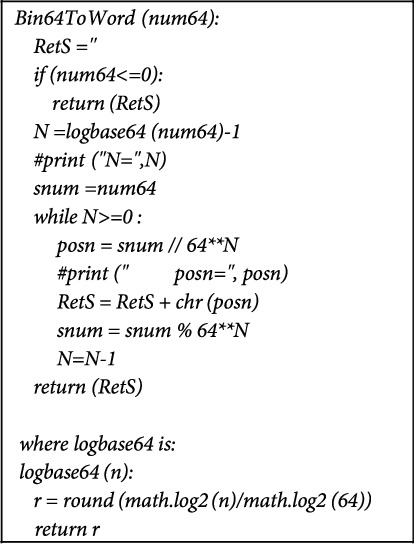
Reverse algorithm to recover a word from its power-of-two representation.

The reverse conversion algorithm that recovers a word text representation from the binary representation is implemented as shown in Figure 6.

## 4. Related Work

Transformers are currently the dominant approach in natural language processing (NLP) [1, 2]. Since their introduction in 2017 [3], they have been successfully applied in various areas of NLP, including semantic key phrase extraction [4], hyperspectral image classification [5], multidimensional essay scoring [6], relation extraction [7], speech recognition [8], sentiment classification [9], geospatial market segmentation [10], fake news detection [11], question answering [12], text summarization [13], and text generation [14]. Good surveys of transformers and related attention technologies can be found in these references [45, 46].

The following paragraphs focus on papers that achieved state-of-art results in NMT using transformers on the WMT2014 data set [15, 47]. The main motivation is to provide an overview of the transformer model evolution over the past few years and the problems addressed by the respective researchers. Most of the models reported accuracies in the English-French data set (EN-FR) and the English-German data set (EN-DE). Note that all accuracies reported in this review are BELU scores. BELU is the standard reliable metric used in evaluating NMT results [48].

In Chen et al. [16], the authors extended local (self) attention with a “syntax distance metric” in an attempt to include all syntactically related source words in the window of the target word. As shown by the model accuracy, the new extended context proved to be more effective in predicting translations. Moreover, the authors experimented with a double context NMT architecture, which consisted of a global context vector and a syntax-directed context vector, which led to better translation accuracy.

In Choi et al. [17], the authors proposed a fine-grained attention mechanism where each dimension of a context vector received a separate attention score instead of a single scalar score. The accuracy obtained was 23.74 BELU for EN-DE.

Liu et al. [18] pointed out that although transformers are very effective in translation, their training is not a trivial task and may destabilize the model and take it off track. Based on the root causes of destabilization, they developed an adaptive model initialization (Admin) approach to stabilize training in the initial phase and release the potential of the model toward the late stages of training. They demonstrated how their Admin approach led to better accuracy and results. The accuracies obtained are 43.8 for EN-FR and 29.03 for EN-DE.

In Lioutas and Guo [19], the authors noted that researchers use either global attention over the whole sequence, which incurs a high time complexity, or local self-attention, resulting in better time complexity, but misses some of the information. Accordingly, they introduced a third type of attention model, which they named “time-aware large kernel (TaLK) adaptive convolution.” TaLK learns to predict the size of a summation kernel instead of using a fixed-sized kernel matrix. This method yielded a better time complexity and led to better accuracy. Accuracies obtained were 43.2 on EN-FR and 29.6 on EN-DE.

In Yang et al. [20], the authors introduced a concerted training NMT framework (CTNMT) that made it possible to integrate pre-trained language models into NMT models. Their proposed CTNMT consisted of three techniques: (a) asymptotic distillation to ensure that the NMT model could retain the previous pre-trained knowledge, (b) a dynamic switching gate to avoid catastrophic forgetting of pre-trained knowledge, and (c) a strategy to adjust the learning pace according to a scheduled policy. The authors showed that their CTNMT model outperformed the baseline model by up to 3 BELU on the English-German WMT-2014 data set and up to 1 BELU on the large English-French model. Accuracies are 42.3 for EN-FR and 30.1 for EN-DE.

In Chen et al. [21], the authors dissected the emerging transformer and similar convolutional seq-to-seq models and tried to map the new innovative features to the traditional RNN model. The result was a new RNN+ model that outperformed the original model on the WMT2014 data set. They also used the new architecture features to develop new hybrid models that beat all previous models on NMT tasks. Accuracies were 41 for EN-FR and 28.49 for EN-DE.

In Clinchant et al. [22], the authors studied how BERT pre-trained models could be exploited for supervised NMT. They experimented with various ways to integrate a pre-trained BERT model with NMT models and studied the impact of the monolingual data used for BERT training on the final translation quality. The data sets used include the WMT2014 English-German, IWSLT15 English-German, and IWSLT14 English-Russian data sets. In addition to standard task test set evaluation, the authors used out-of-domain test sets and noise-injected test sets to assess how BERT pre-trained representations affected model robustness. Their conclusion was that the integration of a pre-trained BERT with NMT is effective. Accuracies were 43.78 for EN-FR and 30.75 for EN-DE.

In Edunov et al. [23], the authors used back translation approaches to augment the training data. Back translation is a simple technique in which additional synthetic parallel data are generated from monolingual target data by training a target-to-source translation system. The data is added to the human training bitext to train the source-to-target system. The authors showed that back-translation led to much better accuracy. The accuracies obtained were 45.6 for EN-FR and 35 for EN-DE.

In Fan et al. [24], the authors analyzed the self-attention (SAN) and feedforward (FFN) components of the transformer model as special cases of a mask attention network (MAN). They showed that SAN and FFN are too special and extreme cases of MAN that did not properly capture attention localness. Accordingly, they introduced a dynamic mask attention network (DMAN), which modified both the SAN and FFN, and proposed a new sequential architecture, DMAN ⟶ SAN ⟶ FFN. Based on their experimentation, the authors reported that the new architecture outperforms the original transformer. The accuracy achieved was 30.4 for EN-DE.

In Imamura and Sumita [25], the authors adapted a monolingually trained BERT in a neural translation model. Instead of using the BERT fine-tuning approach for training, they introduced a two-stage training process that led to better performance, outperforming the original transformer. The accuracy achieved was 28.9 on the EN-DE data set.

In Liu et al. [26], the authors conducted a study to prove the conjecture that very deep transformer models produce much better results than their baseline counterparts. For this purpose, they experimented with a very deep NMT transformer model with up to 60 encoder layers and 12 decoder layers. The results showed that the very deep transformer models outperform their 6-layer baseline counterparts with up to 2 BELUs. The accuracies obtained were 46.4 for EN-FR and 30.1 for EN-DE.

In Liu et al. [27], the authors noted that transformer models lacked the inherent positioning naturally maintained by sequential models. Most transformer models applied artificial positioning schemes that were not very accurate. To solve this problem, the authors developed a new position encoder for transformers. Because the proposed position encoder was based on a free-form flow model (neural ordinary differential equations (ODE)), they named the model FLOATER. Based on their findings, the FLOATER transformer outperformed the baseline transformer by up to 1.1 BELU. The accuracy obtained was 42.7 for EN-FR.

In Maruf et al. [28], the authors noted that although great progress has been made in neural machine translation at the sentence level, current models are still short of producing good translations for full documents. To solve this problem, the authors proposed a scalable top-down hierarchical attention model for context-aware NMT. The model used sparse attention to selectively focus on relevant sentences in the document context and then focused on keywords in those sentences. Finally, the document-level context representation, produced from these attention modules, was integrated into the encoder or decoder of the transformer.

Shaw et al. [29] extended the self-attention mechanism to efficiently consider representations of the relative positions or distances between sequence elements. The accuracy obtained was 41.5 for EN-FR.

In So et al. [30], the authors applied the neural architecture search (NAS) approach to identify a better alternative to the transformer. NAS is an interesting technique that has been recently developed to automate the process of finding the best neural model architecture, given a particular data set. The authors developed “the progressive dynamic hurdle method” to dynamically allocate more resources to candidate models during the search process. The search led to what the authors called the “Evolved Transformer.” Based on the authors' findings, the “Evolved Transformer” model outperformed the original transformer by a small margin. The accuracies obtained were 41.3 for EN-FR and 29.5 for EN-DE.

Takase and Kiyono [31] developed three-parameter sharing strategies for the transformer model: SEQUENCE, CYCLE, and CYCLE (REVERSE). The three-parameter sharing approaches were then compared with the vanilla and UNIVERSAL parameter sharing approaches. Based on the findings of the authors, the proposed strategies achieved BLEU scores comparable to those of the universal approach with a shorter computational time when trained with almost the same parameters for each method. In addition, the proposed strategies slightly outperformed the Universals when spending the same amount of time training them. Accordingly, the proposed strategies were efficient in terms of parameter size and computational time. The accuracy obtained was 40.18 for EN-FR and 35.4 for EN-DE.

In Wu et al. [32], the authors noted that although effective, the dropout regularization mechanism commonly used in neural networks introduced some randomness that led to observable inconsistencies between training and inference. To solve the problem, they developed a simple consistency training strategy to regularize the dropout, R-Drop, which forced consistency between the output distributions of different submodels. According to their results, R-Drop-Transformer outperformed the vanilla transformer by a considerable margin. The accuracies obtained were 43.95 for EN-FR and 30.91 for EN-DE.

In Xu et al. [33], the authors showed that by passing the output (contextualized embeddings) of a tailored and suitable bilingual pre-trained language model named BIBERT as input, an NMT encoder achieved state-of-the-art translation performance. In addition, the authors also introduced a stochastic layer selection approach and a dual-directional translation model to maximize the utilization of contextualized embeddings. The accuracy was 31.26 for EN-DE.

In Yang et al. [34], the authors proposed novel convolutional self-attention networks, which offer self-attention networks (SANs) the ability to strengthen dependencies among neighboring elements and model the interactions between features extracted by multiple attention heads. The accuracy was 31.6 for EN-DE.

Zhang et al. [35] proposed a deep attention model to improve the performance of the encoder-decoder model. They did not clearly specify what sort of attention model they were using. They claimed that the deep attention model outperformed ordinary encoder-decoder models. The accuracies were 39.88 for EN-FR and 26.45 for EN-DE.

In Zhao et al. [36], the authors noted that although transformer self-attention schemes can model extremely long dependencies, the attention in deep layers is overconcentrated on a single token, leading to some problems related to the use of local information. Therefore, the authors developed the parallel multiscale (MUSE) representation model to solve these problems. Based on their findings, MUSE outperformed the original transformer model. The accuracies obtained were 43.5 for EN-FR and 29.9 for EN-DE.

Zhu et al. [37] introduced a method to adapt BERT for use in neural machine translation. Their approach was based on an algorithm named the “BERT-fused model.” In this algorithm, BERT was first used to extract representations for an input sequence. Then, the representations were fused with each layer of the encoder and decoder of the NMT model through an attention mechanism. The accuracies obtained were 43.78 for EN-ER and 30.75 for EN-DE.

## 5. Methods and Materials

Several experiments have been performed to assess the accuracy and robustness of the proposed PIA attention model. All experiments have been performed using the popular WMT 2014 neural machine translation benchmark data set. As will be shown, the experimentation revealed the superiority, robustness, and efficiency of the PIA approach.

### 5.1. The Data Set: The WMT 2014

The experiments below were performed using the WMT-2014 English-French (EN-FR) and English-German (EN-DE) data sets, which are subsets of the European parliament parallel corpus (Europarl). Europarl has been collected from the proceedings of the European Parliament to support statistical machine translation efforts [47]. Since its introduction in one of the challenges in the ninth workshop on statistical machine translation, Euorparl became one of the common benchmarks in the area of neural machine translation (NMT).

The English-French data set is relatively large and consists of 2,007,723 sentence pairs, 55,642,101 English words, 60,125,563 French words, 118,404 distinct English words, and 140,915 distinct French words. The number of benchmark papers published on the data set since 2017 is greater than 50 [15].

The English-German data set comprises 1,920,209 sentence pairs, 53,008,851 English words, 50,486,398 German words, 115,966 distinct English words, and 381,583 distinct German words. The number of published papers on the EN-DE data set is more than 75 [15].

### 5.2. The Model

**Figure 7:**
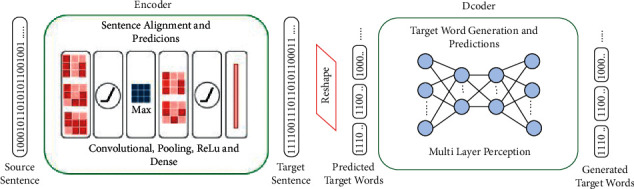
The polynomial regression neural (PRN) model.

The proposed model is a very interesting nonsequential encoder-decoder model, where both the encoder and decoder are simple feedforward models (see Figure 7).

As hinted earlier, the encoder is a neural polynomial regression (NPR) model [38] that takes source sentences as input and applies the neural polynomial regression process to generate predictions for the translation of these sentences.

Both the encoder and decoder work directly on the respective PIA attention vectors from the beginning. The first layer in the encoder is the PIA attention representation of the source sentence (the sentence to be translated). The first layer in the decoder is the predicted PIA representation of the target sentence (the translation) produced by the encoder. The main role of the decoder is to break sentence translations predicted by the encoder into individual words and generate a word-by-word translation.

#### 5.2.1. The Encoder Role

The encoder component is essentially a neural polynomial regression (NPR) [38] feedforward CNN model that aligns the source sentence PIA attention representation with the target sentence PIA attention representation (during training). A side effect of this parallel alignment is a predicted PIA representation of the target sentence (the translation). Note that the predicted target PIA representation is the context vector passed to the decoder.

#### 5.2.2. The Decoder Role

The decoder feedforward MLP model breaks the context vector received from the encoder (which is the predicted PIA representation of the target sentence) into individual words and uses it to generate the word-by-word translations.

#### 5.2.3. Model Parameters

- The encoder is a feedforward CNN model comprising six layers
- The decoder is a feedforward MLP model comprising five layers
- Both the encoder and decoder models apply a cosine similarity accuracy metric combined with the mean squared error cost function

### 5.3. Experimentation

Two rounds of experimentation have been performed: exploration experiments to assess the proposed model at different points in the large data set, followed by full coverage of the data set through five assessment batches. As will be seen, all experiments produced consistent results that widely beat state-of-the-art transformer models.

#### 5.3.1. Exploration: First Rounds of Experiments to Explore Translation Accuracies

The main purpose of this set of experiments is to explore the accuracy, robustness, and scalability of the proposed model. For this purpose, we performed several experiments using selected segments at various points of the NMT-2014 benchmark data sets.

1. First segment: the first 100,000 sentence pairs of the data set
2. Second segment: the middle 100,000 sentence pairs of the data set
3. Third segment: last 100,000 sentence pairs of the data set

These segments have been used to perform three experiments to assess the accuracies of the translations generated by the decoder. Note that in all these experiments, we are assessing the final word-by-word translations generated by the decoder. The types of accuracies assessed are as follows:

- The basic training/test accuracies based on the cosine similarity metric and the mean squared error cost function
- The post-training/testing cosine scores computed by enumerating the cases where the cosine similarity between the predicted and actual translations is greater than 0.5
- The BELU scores computed based on the actual word text translations of the source sentences

Note that the BELU (Bilingual Evaluation Understudy) score [48] is the most important metric in the above set because it is based on the actual word translations, not their numeric representations. Although BELU computation is based on a simple comparison between a hypothesis (the predicted translations) and a reference (the actual translations), the actual process is complex and requires access to full corpora as well as the use of appropriate tokenization strategies [48]. Based on the recommendation of Post [48], the BELU scores have been computed using the SacreBELU library [49, 50], as it has been positively rated by many practitioners.

Three experiments are performed using the abovementioned segments:

- *Experiment 1*: the first segment is divided into training/testing components (70%/30%) and used to compute the respective accuracy metrics. The results are shown in the first row of Table 6. As clear from the table, the basic accuracies and the cosine scores are around 94% and are consistent with each other. The BELU scores are above 73%, a level that has never been reached before.
- *Experiment 2*: The middle segment is used to confirm and verify the results of experiment 1. The basic accuracies and the cosine scores remain around 94%, the BELU scores are above 74%—which is a clear confirmation of the results obtained for the first segment.
- *Experiment 3*: The full middle segment is used for training and the full last segment is used for testing, that is, the testing set in the third case is from a different region of the main data set. In this case, 50% of the combined data set is used for training and 50% for testing. As apparent form, the third row of Table 6, the basic accuracies and the cosine scores remained at the same level (around 94%) in most of the cases, and the BELU score is around 74%. The significance of this experiment is that it shows doubling the size of the set did not lead to significant changes in the accuracies, which indicates the model is accurate and robust and the convergence is fast irrespective of the size of the samples.

#### 5.3.2. Covering the Full Data Set with Five Batches

In the second round of experimentation, the full dataset is divided into 10 equal segments, each segemnt consisting of200,000. rows. The size of the full dataset is approximately 2,000,000 rows. The following five batches have then been created to compute the translation accuracies (decoder outputs):

  Batch 1: train using segment 1, test using segment 5
  Brach 2: train using segment 2, test using segment 6
  Batch 3: train using segment 3, test using segment 7
  Batch 4: train using segment 4, test using segment 8
  Batch 5: train using segment 5, test using segment 10

As apparent from Table 7, the five experiments performed using five batches covering the whole data set did not reveal any significant changes in the accuracies. The maximum test accuracies are 94.21% for the cosine similarity metric, 95.21% for the cosine scores, and 75.07% for BELU scores.

#### 5.3.3. Accuracy for the English-German Data Set (EN-DE)

For completion purposes and because some of the models we are comparing were also applied to the English-German data set, we applied the PIA model to the English-German data set. Using the first 100,000 sentence pairs of the German-English data set. As shown in Tables 8, the basic test accuracy is 94.21%; the test cosine score is 97.49%; and the test BELU score is 66.35%. Clearly, the basic test score is in the same range of the EN-FR data set. The test cosine score is slightly higher than its EN-FR counterpart. The test BELU score is lower by more than 7 points because the value of this parameter highly depends on the language corpora and tokenization approaches. As we will see in the state-of-art comparison tables, this gap in the BELU score is also observable in the transformer results.

#### 5.3.4. Comparison with the State-of-the-Art Transformer Models

Tables 9 and 10 below provide comparisons with the top five scoring transformer modes for the EN-FR (English-French) data set and the EN-DE (English-German) data set, respectively.

It is clear from the tables that PIA is outperforming the top transformer models by more than 65% in the EN-FR case and more than 87% in the case of the EN-DE data set. Given the simplicity and efficiency of the PIA model, this is a great achievement and the best improvement over the past few years.

## 6. Analysis and Discussion

The great breakthrough accuracies produced by the model in Section 5.3 above confirm that PIA is the first model to provide an elegant convincing solution to the NMT sentence alignment problem. Sentence alignment refers to the process used to generate the appropriate translations through the alignment of the source sentence representation with the target sentence representation. The weights of the source attention vector are used to identify which target words should be generated at each decoder output step (teacher forcing).

### 6.1. The Complexity of the NMT Sentence Alignment Process

The main problems with the NMT sentence alignment process include:

- There is no one-to-one correspondence between words in the encoder source sentence and the decoder target sentence.
- There is no guarantee that the order of “source sentence words” and the corresponding “target sentence words” will be the same.
- The length of the translation sentence is in many cases different from the length of the source sentence.
- In many cases, the translation of a full phrase may be just a single word.
- In some cases, some words in the source sentence have no direct translations in the target language, and their meanings are implicitly included in the translations of other words.

The main purpose of NLP attention mechanisms and deep learning embedding approaches [38] is to solve the alignment problem by discovering the implicit semantic dependency rules that associate decoder translations with the respective source words, irrespective of the order and position.

### 6.2. The PIA Solution to the NMT Alignment Problem

Instead of using the classical teacher forcing process, PIA transformed the sentence alignment process into a more reliable neural polynomial regression (NPR) process that produces a full translation for the input sentence in a single step, instead of generating the translation word by word. This full translation is generated by the PIA encoder (Figure 7) in our model.

Since neural polynomial regression is a proven process that generates highly accurate results, it turned out that the predicted target polynomial coefficients are good approximations of the expected translation sentences. Note that since PBPT preserves the positions of words in the source and target sentences, the target polynomial coefficients are directly updated during the NPR process.

The polynomial regression solution is based on the following novel views and ideas:

- Natural language sentences and entities are binary integers in a language-agnostic power-of-two universal numeric language system (UNLS)
- Sentences are transformed into UNLS integers using a simple power-of-two binary polynomial transformation (PBPT)
- Translating a sentence from one language to another is achieved using a simple neural polynomial regression (NPR) model (the encoder in our model) that aligns the UNLS representation of the source sentence with that of the target sentence

### 6.3. The Results Confirm the Expectations and Conjectures

In view of the above analysis, it is not surprising that the simple neural polynomial regression encoder produced near-perfect predictions of full translations of the source sentences. It is also not surprising that the convergence is fast, and the accuracies are consistent. The only job of the decoder is to decompose the full sentence translations produced by the decoder into individual target word translations. This is a straightforward process since the positions of words are preserved in target sentences.

The above experiments provided concrete evidence that PIA produces consistently excellent cosine and BELU scores under different conditions and circumstances. It has also been observed that the accuracies remain consistent under different training and test sizes. Another important fact is that all the above results are obtained using only 10 epochs of training on normal laptops without GPUs or TPUs, and it took only a few minutes to complete the training and testing.

These interesting results confirm our earlier reasoning that the alignment of the PIA power-of-two sentence representations is near-perfect. This is mainly because these representations are unique fingerprints that accurately capture the spatial dependencies between words in sentences, as well as the semantic context for each word in the sentence. This is achieved using strict power-of-two number rules. After all, a sentence is just a number in a power-of-two number system.

It is felt that this approach is quite promising, and we are just touching the tip of the iceberg.

One promising direction is to investigate the possibility of developing a power-of-two algebraic framework for performing challenging NLP tasks.

## 7. Conclusion

This paper presented a novel polynomial inherent attention (PIA) model that outperforms state-of-the-art transformer models by a wide margin. The model is based on a simple power-of-two parallel positioning numeric transformation that transforms sentences and words into unique binary integers in some power-of-two number systems. PIA provides excellent solutions to NMT alignment and positioning problems and reduces the machine translation process to a simple polynomial regression problem [38] (fitting one polynomial to another). As expected, the accuracy and BELU scores are high, and the model outperforms all known attention models by a wide margin. The BELU scores provided by the model on the WMT-2014 range from 73 to 75 BELU for the EN-FR component (65.7% improvement over the best transformer results) and 66.35 BELU for the EN-DE German set (87.42% improvement over the best transformer result). The BELU scores have been computed using SacreBELU [49, 50]. To improve and streamline the translation process, a neural translation dictionary (NTD) is being constructed [56]. The main purpose of the NTD is to improve the prediction process and further study PIA behavior and features. Certainly, the approach is very promising, and more work is being done to investigate the possibilities.

## Figures and Tables

**Table 1:** PBPT sentence representation in terms of polynomial coefficients.

*wint*_0_	*wint*_1_	*wint*_2_	…	*wint*_*n*_

**Table 2:** Power -of-two representation of the word “computer”.

		c	o	m	p	u	t	e	r
000000	000000	000011	001111	001101	010000	010101	010100	000101	010010

**Table 3:** Power-of-two representation of the word “science”.

			s	c	i	e	n	c	e
000000	000000	000000	010011	000011	001001	000101	001010	000011	000101

**Table 4:** Power-of-two representation of the word “department”.

d	e	p	a	r	t	m	e	n	t
000100	000101	010000	000001	010010	010100	001101	000101	001010	010100

**Table 5:** Power-of-two representation of the full sentence “computer science department”.

Computer	Science	Department
000000 … 010010	000000 … 000101	000100 … 010100

**Table 6:** Exploring decoder translation accuracies using three select segments.

Sample	Train acc (%)	Test acc (%)	Train cosine score (%)	Test cosine score (%)	Train BELU score (%)	Test BELU score (%)
First segment	93.86	93.80	93.96	94.19	73.05	73.10
Middle segment	94.18	94.09	94.53	94.17	74.54	74.23
Train by mid/test by last	94.14	93.80	96.06	94.50	76.18	74.08

**Table 7:** Results for the full data set covered by five Batches.

Batch	Train acc (%)	Test acc (%)	Train cosine score (%)	Test cosine score (%)	Train BELU score (%)	Test BELU score (%)
1	93.88	94.21	93.90	95.15	73.01	75.07
2	93.79	94.10	93.98	94.89	73.11	73.32
3	93.99	93.93	95.46	95.15	74.93	74.95
4	93.98	93.78	96.03	95.21	75.88	74.77
5	92.08	92.06	93.92	93.84	75.06	74.08

**Table 8:** Results for the English-German (EN-DE) data set.

Train acc (%)	Test acc (%)	Train cosine score (%)	Test cosine score (%)	Train BELU (%)	Test BELU (%)
94.6	94.21	97.86	97.49	66.05	66.35

**Table 9:** Comparison of PIA with the top state-of-the-art models (English-French).

Rank	Model	BELU score	Paper
1	Transformer + BT-(ADMIN init)	46.4	Liu et al. [26]
2	Noisy back-translation	45.6	Edunov et al. [23]
3	mRASP + Fine-Tune	44.3	Xiao Pan et al. [51]
4	Transformer + R-Drop	43.95	Wu et al. [32]
5	Transformer (ADMIN init)	43.8	Liu et al. [26]
Top	PIA (our model)	**75.07**	This paper

**Table 10:** Comparison of PIA with the top five state-of-the-art models (English-German).

Rank	Model	BELU score	Paper
1	Transformer Cycle Rev	35.14	Takase and Shun [52]
2	Noisy back-translation	35.0	Edunov et al. [11]
3	Transformer+ Rep Uni	33.89	Takase and Kiyono [53]
4	T5-11B	32.1	Raffel et al. [54]
5	BiBERT	31.26	Xu et al. [55]
Top	PIA (our model)	**66.35**	This paper

## Data Availability

The WMT-2014 machine translation data set is publicly available and has been used by many researchers in the area of machine translation: link 1, Europarl Parallel Corpus (https://statmt.org); link 2, Translation Task-ACL 2014 Ninth Workshop on Statistical Machine Translation (https://statmt.org).
